# Aging, prevalence and risk factors of MRI-visible enlarged perivascular spaces

**DOI:** 10.18632/aging.204181

**Published:** 2022-07-15

**Authors:** Frances Rodriguez Lara, Ashlea Lynn Scruton, Adlin Pinheiro, Serkalem Demissie, Pedram Parva, Andreas Charidimou, Michael Francis, Jayandra J. Himali, Charles DeCarli, Alexa Beiser, Sudha Seshadri, Jose R. Romero

**Affiliations:** 1 Boston University School of Medicine, Boston, MA 02118, USA; 2 NHLBI’s Framingham Heart Study, Framingham, MA 01702, USA; 3 Department of Biostatistics, Boston University School of Public Health, Boston, MA 02118, USA; 4 Department of Radiology, Veterans Affairs Boston Health System, Boston, MA 02130, USA; 5 Department of Neurology, Boston University School of Medicine, Boston, MA 02118, USA; 6 The Glenn Biggs Institute for Alzheimer's and Neurodegenerative Diseases, University of Texas Health Sciences Center, San Antonio, TX 78229, USA; 7 Department of Neurology, University of California at Davis, Davis, CA 95817, USA

**Keywords:** cerebral small vessel disease, neurological markers, aging, disease marker, perivascular spaces

## Abstract

Background and purpose: Cerebral small vessel disease (CSVD) increases with age and is associated with stroke and cognitive decline. Enlarged Perivascular Spaces (ePVS) is an emerging marker of CSVD, but its prevalence over the life span remain unclear. We characterized the age and sex-specific prevalence of ePVS and relation to age-specific risk factors, in a large community-based sample.

Methods: We included 3,710 Framingham Heart Study participants with available brain MRI (average age 61.4±14.6, 46% men). ePVS burden was rated in the centrum semiovale (CSO) and basal ganglia (BG) regions. Individual vascular risk factors were related to ePVS burden in the CSO, BG, and mixed CSO-BG regions using multivariable adjusted ordinal logistic regression analysis.

Results: Severe ePVS prevalence increased with age in men and women, and paralleled increase in vascular risk factors, and prevention treatment use. Older age, hypertension (and resulting higher treatment use), higher systolic and diastolic blood pressure, and smoking were associated with higher burden of ePVS in the CSO, BG and mixed regions.

Conclusions: Our observations reinforce the hypothesis that ePVS may be a marker of aging-driven brain vascular pathologies, and its association with vascular risk factors support their role as CSVD imaging biomarker.

## INTRODUCTION

The aging process in the brain is characterized by progressive degenerative changes, some of which play major roles in diseases that affect primarily elderly, such as stroke and dementia. Impaired perivascular drainage and cerebral small vessel disease are two of such changes. The recently recognized brain glymphatic system is formed by perivascular spaces that represent drainage routes for cerebral metabolites, such as beta amyloid (Aβ), playing a critical role in cerebral homeostasis [1]. It is believed that when perivascular spaces (PVS) are dilated, they become visible on conventional structural brain MRI and considered to represent dysfunction of the perivascular drainage and cerebral small vessel disease. Enlarged perivascular spaces (ePVS) can be detected and quantified using brain MRI, and have recently emerged as subclinical markers of risk for cognitive impairment, dementia and stroke [2–4].

A higher burden of ePVS is considered to signal ongoing neurodegeneration and microvascular injury, [5] both of which are also recognized factors in the process of aging. While the underlying pathophysiology of ePVS may vary depending on the sample studied and remains to be elucidated, it has been suggested that the most common sporadic forms of cerebral small vessel disease (i.e. cerebral amyloid angiopathy and hypertensive angiopathy) may relate to ePVS burden. Similar to other MRI biomarkers, the predominant spatial distribution of ePVS burden, may reflect distinct mechanisms: lobar (centrum semiovale) distribution is likely related to cerebral amyloid angiopathy (CAA), deep (basal ganglia) distribution likely caused by hypertensive vasculopathy, and mixed location reflecting the interplay of both conditions, or the predominant effect of one over the other [6]. Such anatomical distinctions have been first observed in ePVS in spontaneous intracerebral hemorrhage patient cohorts [7]. However, further insight is needed about changes in ePVS burden across age groups, and their relation to vascular risk factors across the same age groups in unselected healthy elderly population-based samples.

In this report we aim to describe 1) the age and sex specific prevalence of ePVS in a large sample of asymptomatic, community dwelling individuals, and contrast ePVS prevalence with the prevalence of vascular risk factors in the same age groups, and 2) study the association of vascular risk factors with burden of ePVS by brain region. This knowledge will help support the increasing number of studies of ePVS as a biomarker of aging and age related adverse neurological outcomes.

## MATERIALS AND METHODS

### Study sample

The Framingham Heart Study is a prospective, multigenerational, population-based cohort study that began with enrollment of the Original cohort of 5,209 participants in 1948. These participants have been examined approximately once every 2 years. The Offspring Cohort of 5,124 participants recruited in 1971 included offspring of the Original Cohort and their spouses and has been examined approximately every 4 years. The Third Generation (Gen 3, n=4,095) and the New Offspring (NOS, n=103) cohorts were recruited in 2002 and has been examined three times. Since the first three generations of the FHS participants were predominantly of white European descent, the Heart Study enrolled an OMNI cohort in 1994 that includes 506 individuals of African American, Hispanic, Asian, Indian, Pacific Islander and Native American descent.

Since 1999 participants have been invited to undergo brain MRI. The present study included participants from all cohorts with available brain MRI acquired between 2000 and 2015. Participants who underwent brain MRI attended a baseline clinical examination between 1997 and 2014 (25^th^-31^st^ examination cycle for Original cohort participants, 7^th^-9^th^ examination cycle for Offspring participants, 1^st^-2^nd^ exam cycles for NOS/Gen 3 participants, and 2^nd^-4^th^ exams for OMNI 1 participants).

**Figure 1:**
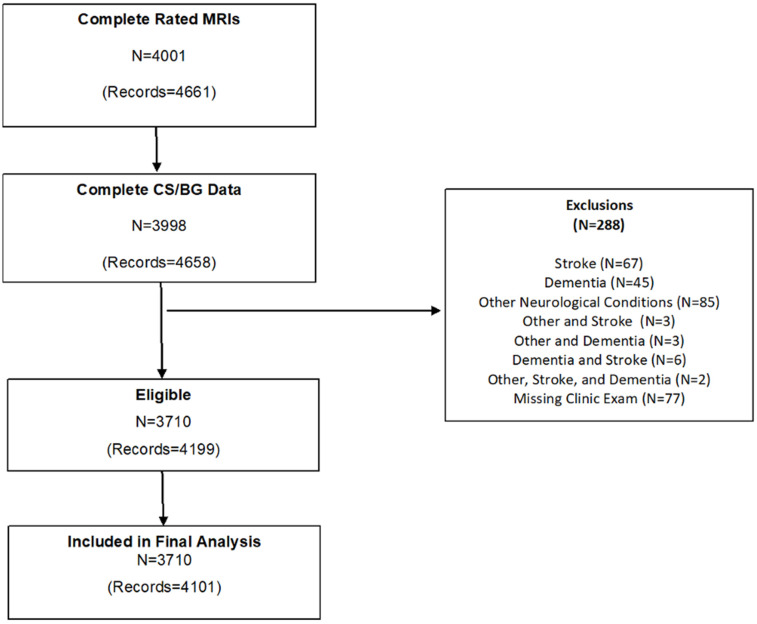
Flow chart of study sample selection.

The flow chart of the sample selection is described in Figure 1. In total, there are 10,589 MRI scans from 5,594 FHS participants, of which 4,658 MRI scans from 3,998 participants had ePVS ratings. Exclusion criteria for the present study included refusal or contraindication for MRI (pacemaker or other implantable devices, metallic foreign body, claustrophobia), scans with significant artifacts precluding ePVS assessment, scans without a corresponding clinic examination, and history of clinical stroke, dementia, and neurological conditions that could affect brain MRI measurements (such as head trauma, multiple sclerosis, brain tumor). Following these exclusions the sample included 4,199 scans from 3,710 participants.

3,377 participants only had one MRI scan. For the 822 participants who had multiple scans, we selected one scan corresponding to a unique primary clinic exam. If no primary clinic exam was assigned, we selected the latest scan within the exam cycle such that our final sample included one scan from each available exam cycle.

The final sample included 4,101 scans (270 Original cohort, 1814 Offspring, 19 NOS, 1848 Third Gen, 149 OMNI 1) from 3,710 participants. The Institutional Review Board of Boston University Medical Center approved the study protocol and informed consent was obtained from all subjects.

### Brain MRI

### Acquisition and analysis

Brain MRI acquisition measures and image processing methods have been described in detail [8, 9]. Brain MRI were acquired using a 1T (1999–2005) or 1.5T (after 2005) Magnetom scanner (Siemens Medical, Erlangen, Germany).

### Enlarged perivascular spaces (ePVS)

The MRI characteristics of ePVS were based on prior consensus criteria by the Standards for Reporting Vascular Changes on Neuroimaging Criteria (STRIVE consortium) [10]. Briefly, ePVS met the following criteria: signal intensity similar to CSF on all sequences, adherence to the course of penetrating vessels, linear (parallel to the penetrating vessel) or round/ovoid (perpendicular to the penetrating vessel), and a diameter smaller than 3mm.

### ePVS rating measures

**Figure 2:**
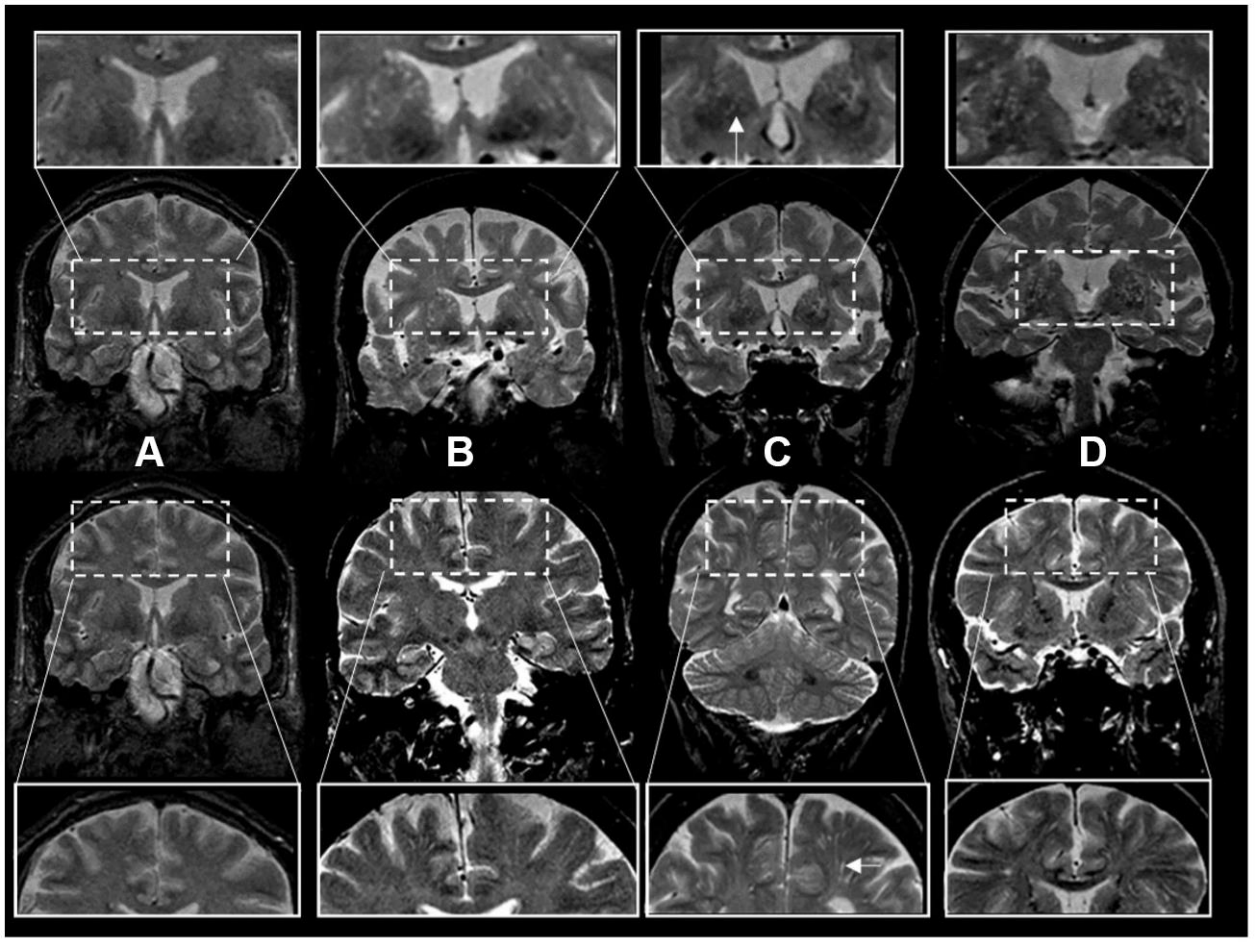
**Enlarged perivascular space burden using ratings in coronal T2-weighted MRI sequences.** *Top rows: basal ganglia region. Bottom rows: centrum semiovale region. The inserts represent a closer view. White arrows point to individual examples of enlarged perivascular spaces (panel C inserts). †Grades of ePVS burden are based on T2 weighted coronal views. (**A**) Grade I (0-10), (**B**) Grade II (10-20) (**C**) Grade III (20-40) (**D**) Grade IV (40+).

Scans were rated by three investigators (JRR, PP, AS) blinded to the subjects’ demographic and clinical information. T2-weighted axial MRI sequences were the main sequence used for rating ePVS following a validated method [11]. Brain topography of ePVS was classified as centrum semiovale (CSO) and basal ganglia (BG). We also analyzed a mixed group including ePVS in both regions. The burden of ePVS in each region was categorized into grades based on ePVS counts: grade I (1-10), II (11-20), III (20-40) and IV (>40). In a subset of legacy scans from an older dataset, coronal acquisitions were of higher resolution than axial views and were used for ratings (N=1122). For these ratings using coronal views to approximate ratings using axial views we assessed the entire BG region in all slices above the anterior commissure and below the level of the roof of the lateral ventricles. Ratings were based on ePVS burden in the BG bilaterally or in case of presence of large incidental lesions (such as large covert infarcts – without corresponding clinical stroke or TIA event) in the contralesional side. Ratings of the CSO region were performed evaluating the corona radiata, above the level of the lateral ventricles, and subcortical white matter. We categorized burden of ePVS using the same categories as in axial views (Figure 2). In a subset of scans with available high-resolution axial and coronal reconstructions, we compared ePVS ratings in BG and CSO regions based on axial versus coronal sequences. ePVS ratings in BG and CSO were done on 20 scans rated in random order in two separate occasions using axial sequences first, and repeating ratings with the coronal sequence after changing scan order randomly and blinded to the axial ratings. Ratings in axial and coronal sequences were highly correlated with an intraclass correlation (ICC)=0.91.

### ePVS ratings reliability measures

Intra-rater reproducibility was assessed for each rater, using 200 scans (JRR, PP) or 20 scans (AS), on two separate occasions 2 to 4 weeks apart, and changing randomly the order of scans between the two reading sessions. Inter-rater reproducibility measures were compared between the primary rater (JRR) and secondary raters (PP, AS) using 200 scans (JRR vs PP), and 20 scans (JRR vs AS). The intra-rater reliability was good to excellent (ICC basal ganglia JRR and PP=0.76, AS=0.74; centrum semiovale JRR= 0.81, PP=0.76, AS=0.81). Inter-rater reliability comparing two independent readers was excellent (ICC basal ganglia JRR vs PP = 0.86, JRR vs. AS =0.83; centrum semiovale JRR vs. PP= 0.8, JRR vs. AS= 0.90). For EPVS ratings using coronal sections we observed excellent intra-rater reproducibility (ICC=0.9).

### Additional brain MRI markers of cerebral small vessel disease

We also used existent ratings of other CSVD markers based on brain MRI to evaluate the relation of ePVS and other CSVD markers. White matter hyperintensities volume (WMHV), total cranial and brain volumes were rated on FLAIR and T1 weighted MRI sequences respectively, using quantitative analyses as previously reported [12, 13]. We used the total cranial to brain volume (TCBV) ratio to account for head size differences. Presence of cerebral microbleeds (CMB) were rated on T2*weighted sequences using previously published guidelines [14, 15]. Covert brain infarcts (CBI) were determined according to STRIVE criteria [10, 16].

### Vascular risk factors

Systolic (SBP) and diastolic (DBP) blood pressures (mmHg) were each taken as the average of the Framingham clinic physician’s two measurements. Hypertension status was evaluated using the JNC-7 criteria (SBP ≥140 mmHg or DBP≥90 mmHg or use of antihypertensive medications). In addition, hypertension categories were assessed using SBP and DBP measurements, regardless of anti-hypertensive use: normal (SBP <120 mmHg and DBP <80 mmHg), pre-hypertensive (SBP 120-139 mmHg or DBP 80-89mmHg), and hypertensive (SBP ≥140 mmHg or DBP ≥90 mm Hg).

Diabetes was defined as a random blood glucose ≥200 mg/dl (≥11.1 mmol/L) for the Original cohort, fasting glucose ≥126mg/dl (≥7 mmol/L) for the Offspring, Third Generation, NOS, and OMNI 1 cohorts, or use of insulin or oral hypoglycemic medications (for all cohorts).

Total cholesterol, high-density lipoprotein (HDL) cholesterol, and triglycerides (mg/dL) were measured on fasting specimens in the Offspring cohort, and random samples in the Original cohort. LDL-cholesterol concentrations (mg/dL) were calculated according to the Friedewald equation: LDL= (TC – HDL + (TG /5), where LDL = low density lipoprotein, TC = total cholesterol, HDL=high density lipoprotein, TG= triglycerides. Because the formula is unreliable for individuals with TG levels above 400mg/dL, participants whose triglyceride levels were above 400mg/dL had missing LDL values.

Body Mass Index (BMI) was measured by finding the ratio of the participants’ weight and squared height (kg/m^2^). Participants were classified as underweight (BMI < 18.5 kg/m^2^), normal (BMI 18.5-24.9 kg/m^2^), overweight (BMI 25.0-29.9 kg/m^2^), and obese (BMI > 30.0 kg/m^2^), as defined by the National Institutes of Health.

### Statistical analysis

The main outcomes of interest in our analyses were CSO ePVS, BG ePVS, and a mixed CSO-BG score reflecting high burden ePVS in either region or both. High ePVS burden was defined as counts greater than 20 (i.e. grades III or IV) within each region. The CSO-BG mixed score denoted the number of regions with high ePVS burden (0=none, 1=one region, or 2=both regions). CSO and BG ePVS (grades I-IV) and the CSO-BG mixed score (0-2) were each treated as ordinal outcomes.

Descriptive statistics of demographic variables and risk factors are described for the overall sample by brain region using means and standard deviations for continuous variables and frequencies and relative frequencies for categorical variables. Prevalence of ePVS in men and women by 10-year age intervals are presented in bar charts for each region. Continuous vascular risk factors were standardized to mean zero and standard deviation of one and described in bar charts for the overall sample by 10-year age intervals. We also provide descriptive statistics for the relation of ePVS burden and other brain MRI CSVD markers (WMHV, TCBV ratio, CMB, and CBI).

Ordinal logistic regression analysis was used to calculate odds ratios and 95% confidence intervals for the association between each individual vascular risk factor and each of the following outcomes: CSO only ePVS, BG only ePVS, and mixed CSO-BG ePVS. Odds ratio estimates for SBP, DBP, total cholesterol, LDL, HDL, and BMI represent a one-standard deviation change in the proportional odds of ePVS. We used empirical (robust) standard error estimates to account for dependence, but potentially because of the relatively small number of repeated scans, the results were similar to those obtained using classical standard errors.

Models were adjusted for age at MRI, sex, FHS cohort, and time interval between risk factor measurement and MRI acquisition. Since the NOS and Third Generation cohorts had the same enrollment periods, nineteen participants in the NOS cohort were combined into the Third Generation cohort for analysis. We also adjusted for the MRI sequence used for ratings (axial vs. coronal) but no significant differences were noted.

All analyses were performed using Statistical Analyses System (SAS) software version 9.4 (SAS Institute, Cary, NC). A two-sided p-value <0.05 was considered statistically significant.

## RESULTS

### Age and sex specific prevalence of ePVS and vascular risk factors

**Figure 3:**
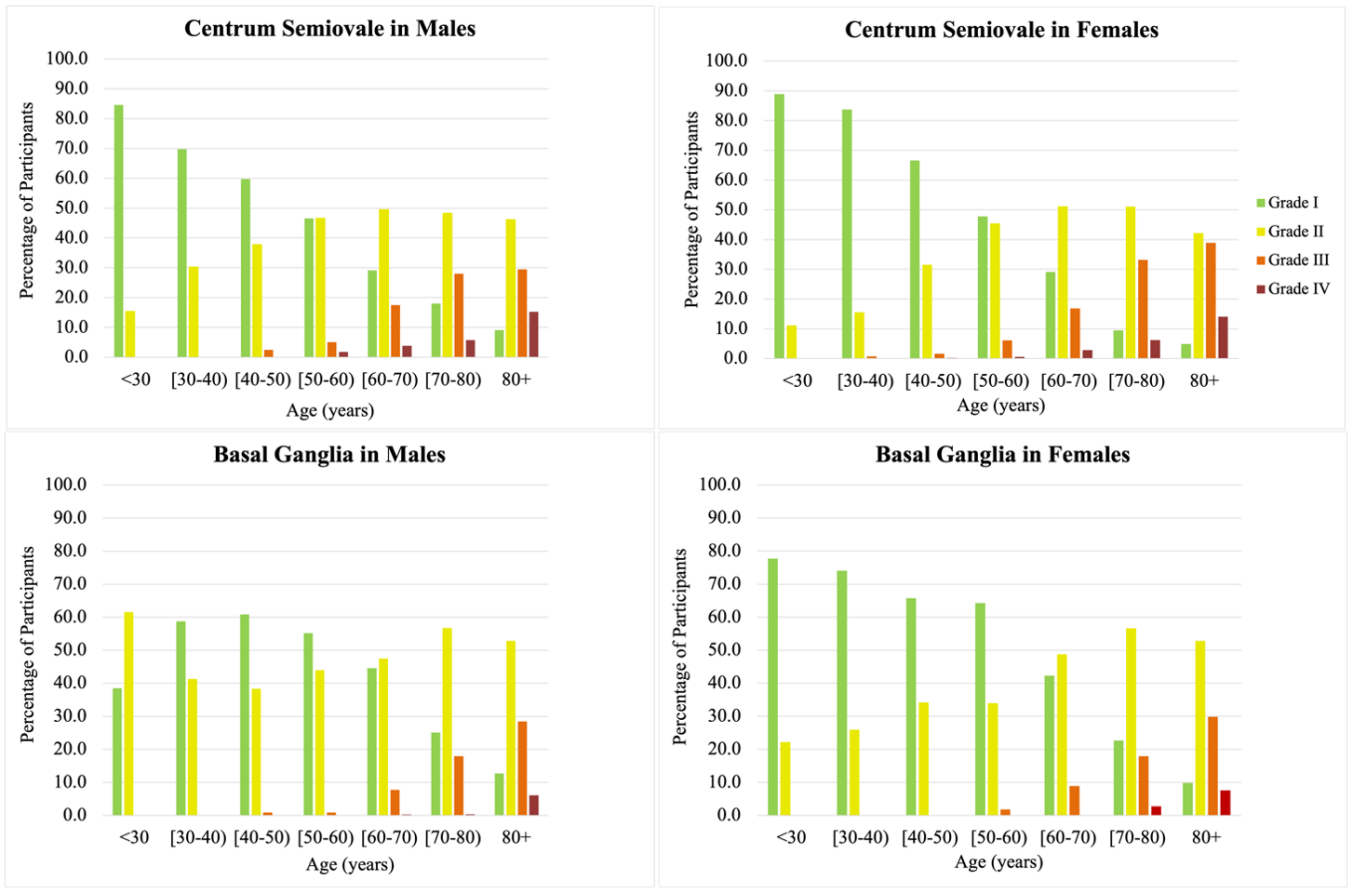
**Age and sex-specific prevalence of enlarged perivascular spaces (ePVS) stratified by brain topography (basal ganglia and centrum semiovale).** Notice the increase prevalence of high burden ePVS (grades III and IV, orange and red color bars) with age starting around 50-60 years, and the decreasing prevalence of low burden ePVS (grades I and II, green and yellow bars) as age increases from younger age groups, becoming uncommon in the elderly.

Baseline demographic characteristics of our sample by ePVS grade in each brain region are shown in Table 1. The prevalence of high ePVS burden increased in both brain regions as average age increased. High burden ePVS (grades III and IV) was low in participants younger than 60 years old, less than 10% in the BG and CSO (Figure 3). In contrast, most participants over age 80 had high ePVS burden in both the BG and CSO regions, with predominance in the CSO. No substantial differences were observed in the prevalence of high ePVS burden across brain regions between men and women throughout the life span, although in the overall sample a smaller proportion of males was noted in participants with grade IV ePVS in the BG (29% men, 61% women).

**Table 1:** Sample characteristics in the overall sample and stratified by brain region and ePVS burden.

		**Centrum Semiovale (CSO)**				**Basal Ganglia (BG)**			
	**All** **N=4101**	**Grade I** **(N=1521)**	**Grade II** **(N=1791)**	**Grade III** **(N=629)**	**Grade IV** **(N=159)**	**Grade I** **(N=1836)**	**Grade II** **(N=1844)**	**Grade III** **(N=372)**	**Grade IV** **(N=48)**
***Clinical Characteristics***									
Men	1873 (46)	708 (47)	835 (47)	256 (41)	73 (46)	825 (45)	878 (48)	156 (42)	14 (29)
Age at MRI, years	60 (15)	53 (12)	63 (13)	73 (10)	77 (11)	55 (12)	64 (14)	77 (9)	83 (7)
***Vascular risk factors***									
Systolic blood pressure, mm Hg	124 (18)	117 (14)	125 (18)	131 (18)	137 (20)	119 (15)	126 (18)	136 (19)	136 (18)
Diastolic Blood pressure, mm Hg	73.0 (10)	74 (9)	73 (10)	71 (10)	73 (11)	73 (9)	73 (10)	72 (10)	69 (11)
Hypertension Category									
Normotensive‡	1676 (41)	829 (55)	653 (36)	163 (26)	30 (19)	915 (50)	683 (37)	70 (19)	8 (17)
Prehypertension‡	1692 (41)	571 (38)	781 (44)	278 (44)	62 (39)	730 (40)	779 (42)	162 (44)	21 (44)
Hypertension‡	733 (18)	121 (8)	357 (20)	188 (30)	67 (42)	191 (10)	382 (21)	140 (38)	19 (40)
Hypertension Stage 1 or higher §	1722 (42)	354 (23)	850 (48)	404 (64)	114 (73)	530 (29)	881 (48)	270 (73)	40 (83)
Current smokers	307 (7)	121 (8)	132 (7)	42 (7)	12 (8)	137 (7)	151 (8)	18 (5)	1 (2)
Total Cholesterol (mg/dL)	188 (36)	187 (33)	189 (38)	191 (37)	188 (36)	189 (34)	188 (38)	189 (34)	183 (39)
HDL (mg/dL)	60 (18)	60 (18)	59 (18)	60 (19)	58 (19)	60 (18)	59 (18)	61 (20)	58 (17)
LDL (mg/dL)	105 (31)	105 (29)	106 (32)	106 (32)	104 (28)	106 (31)	106 (32)	103 (29)	99 (32)
Triglycerides (mg/dL)	118 (74)	111 (74)	119 (74)	128 (67)	127 (88)	114 (73)	119 (75)	128 (68)	131 (77)
Fasting plasma glucose (mg/dL) †	100 (22)	97 (18)	102 (23)	104 (24)	108 (29)	98 (19)	103 (25)	102 (16)	105 (20)
Non-fasting plasma glucose (mg/dL) †	114 (42)	-	118 (46)	111 (37)	112 (46)	121 (45)	117 (44)	110 (41)	98 (18)
Diabetes mellitus||	411 (10)	82 (5)	218 (13)	89 (15)	22 (16)	130 (7)	228 (13)	44 (13)	9 (23)
Body Mass Index	27.9 (5)	28 (5)	28 (5)	27 (5)	27 (5)	28 (5)	28 (5)	28 (5)	27 (5)
Body Mass Index Category									
Underweight	40 (1)	17 (1)	15 (1)	4 (1)	4 (3)	13 (1)	21 (1)	5 (1)	1 (2)
Normal	1225 (30)	481 (32)	489 (28)	205 (34)	50 (33)	569 (31)	524 (29)	115 (33)	16 (36)
Overweight	1626 (40)	611 (40)	718 (40)	235 (38)	61 (40)	736 (40)	744 (41)	128 (36)	18 (40)
Obese	1170 (29)	410 (27)	556 (31)	167 (27)	37 (24)	513 (28)	542 (30)	105 (30)	10 (22)
Lipid lowering medication Use	1206 (29)	311 (20)	596 (33)	239 (38)	60 (38)	410 (22)	613 (33)	157 (42)	26 (54)
Antihypertensive Use	1405 (34)	285 (19)	701 (39)	331 (53)	88 (56)	421 (23)	720 (39)	227 (61)	36 (75)

Values represent mean (SD) for continuous variables and n (%) for categorical variables.

†Random blood glucose was used for the Original cohort.

‡Normal blood pressure is defined as systolic blood pressure (SBP) <120 mmHg and diastolic blood pressure (DBP) <80 mmHg. Prehypertension is defined as SBP of 120-139 mmHg or DBP 80-89mmHg. Hypertension is defined as SBP ≥140 mmHg or DBP ≥90 mm Hg. Blood pressure categories are defined irrespective of hypertension treatment status.

§Hypertension Stage 1 or higher is defined as hypertension or antihypertensive medication use.

| |Missing risk factor data: diabetes (N=152), LDL (N=100).

**Figure 4:**
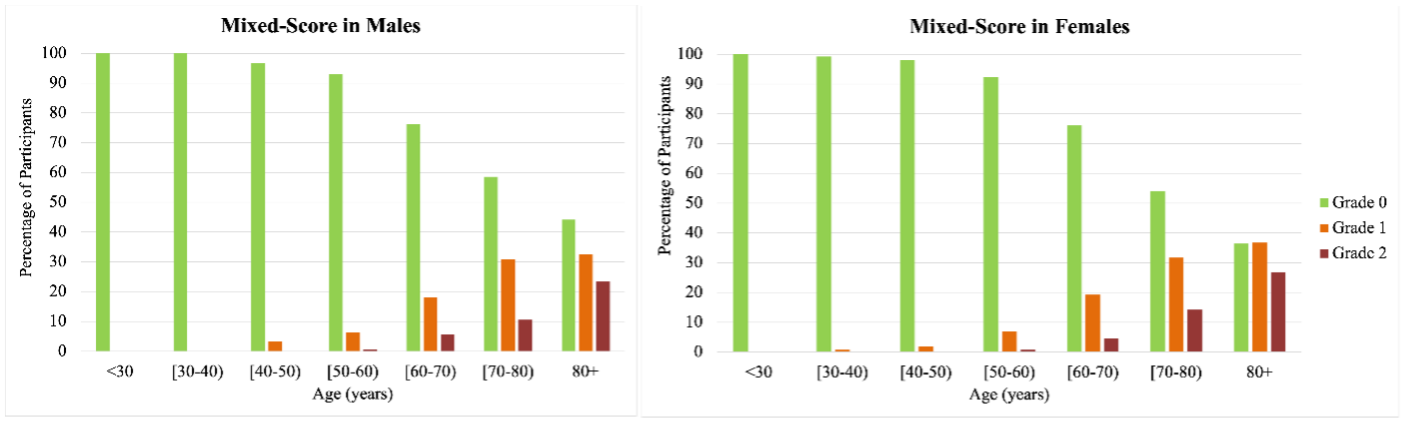
**Age- and sex-specific prevalence of mixed (CSO and/or BG) high burden enlarge perivascular spaces.** High ePVS burden was defined as grades III and IV within each region. The mixed score reflects participants with no high ePVS burden in neither the Basal Ganglia or Centrum Semiovale (grade 0, green color bar), high ePVS burden in either the Basal Ganglia or the Centrum Semiovale (grade 1, orange color bar), and high burden in both the Centrum Semiovale and the Basal Ganglia (grade 2, dark red color bar). Notice that high burden in both regions increases with age, beginning in the 50-60 age group.

In our sample, the age at which high burden ePVS in either brain region was first detected was between 50 and 60 years (Figure 3). While ePVS were visible in all scans, the prevalence of severe ePVS in the BG, CSO and mixed brain regions increased steadily by age group for both men and women (Figures 3, 4). Overall, inspection of the data did not show meaningful differences between men and women. Among scans of participants 80 years of age and over, 50% had high ePVS burden (grade III-IV) in the CSO region and 36% in the BG region.

**Figure 5:**
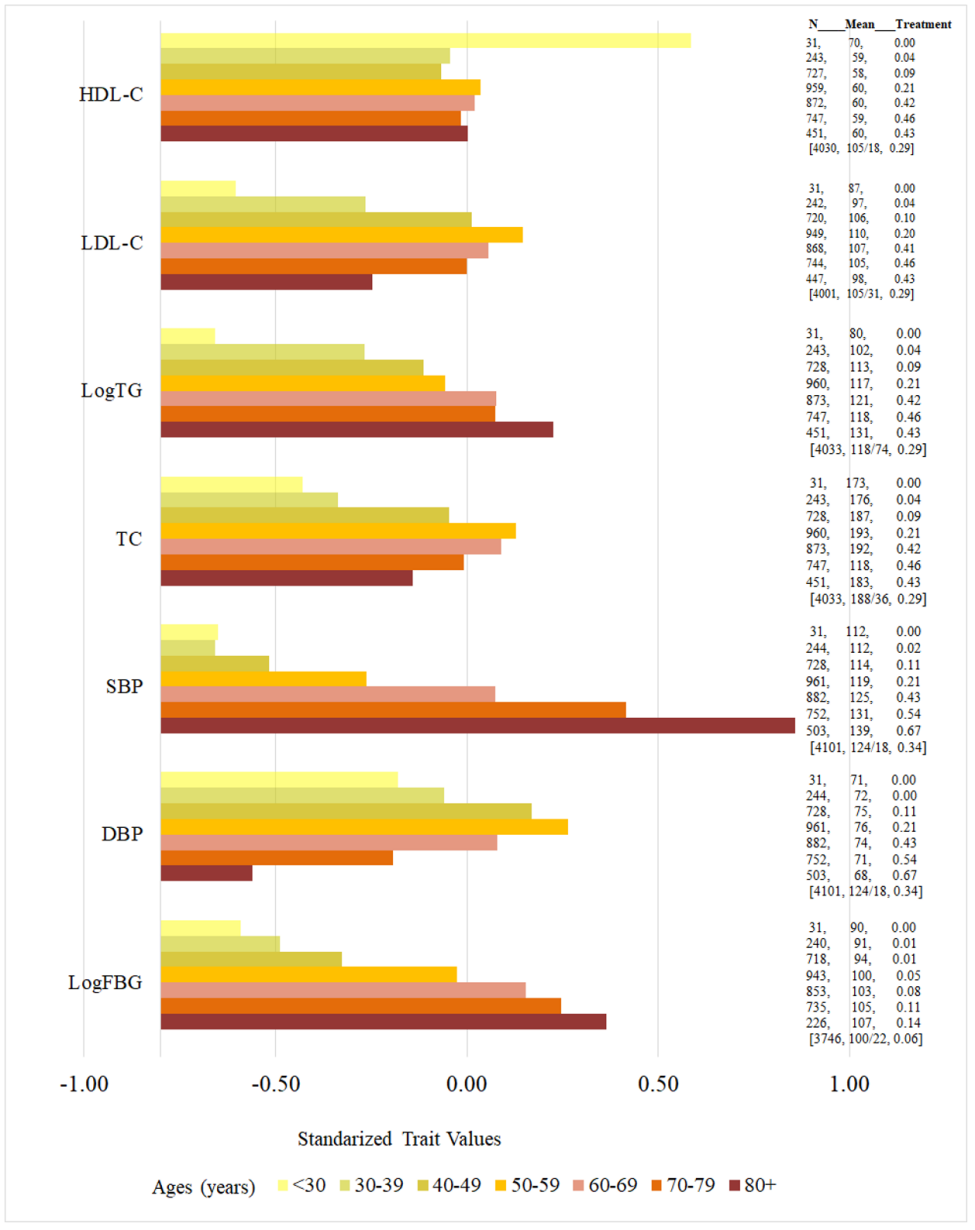
**Age-specific vascular risk factors (standardized values) and proportion of vascular risk factor treatment use.** High-Density Lipoprotein-Cholesterol (HDL-C, mg/dL), Low-Density Lipoprotein-Cholesterol (LDL-C, mg/dL), total glucose (TG, mg/dL), total cholesterol (TC, mg/dL), systolic blood pressure (SBP, mmHg), diastolic blood pressure (DBP, mmHg), fasting blood glucose (FBG, mg/dL). Horizontal color bars represent standardized values for each vascular risk factor, presented by age group in 10-year strata, from younger to older groups (top to bottom). The three columns on the right of the bars present the following data for each age group: number of individuals in each age stratum (N), mean value for each vascular risk factors in units used in clinical practice (unstandardized, middle column), and proportion of individuals using medication for the treatment of the respective risk factor (percent, third column); square brackets were used for the entire sample [n, unstandardized mean/standard deviation (SD), percent of individuals using medication]. Notice that the number of participants with each risk factor increases progressively as age group increases until middle age, where some of the risk factors plateau or decrease, which parallels an increase in the percent of individuals using medications for the treatment of the individual risk factor. However, some risk factors rise across all age groups despite medication use increases, including SBP, TG and FBG.

**Figure 6:**
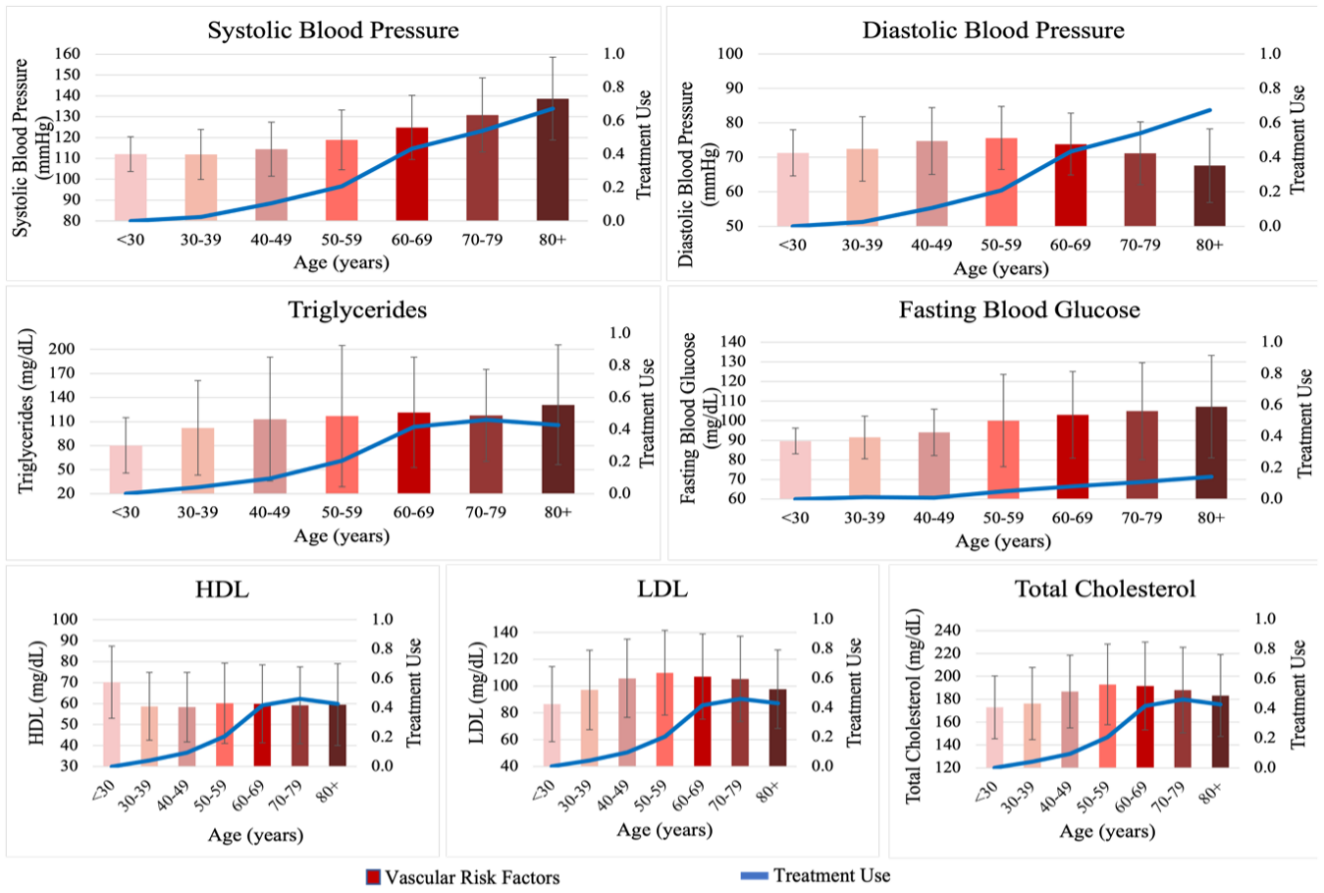
**Levels of vascular risk factors and proportion of treatment use across age groups during the entire adult life span.** Vascular risk factors and treatment use stratified in 10-years of age groups. High-Density Lipoprotein-Cholesterol (HDL-C, mg/dL), Low-Density Lipoprotein-Cholesterol (LDL-C, mg/dL), total glucose (TG, mg/dL), total cholesterol (TC, mg/dL), systolic blood pressure (SBP, mmHg), diastolic blood pressure (DBP, mmHg), fasting blood glucose (FBG, mg/dL). The red bars represent mean levels and the error bars represent standard deviation of vascular risk factors in units commonly used in clinical practice across age groups by decade, during the entire adult life span. The blue line represents the proportion of participants using medications for the respective risk factor. Notice the increasing levels of lipids and blood glucose from early to mid-adult life, followed by plateau or decrease in late life, which is also paralleled by increasing prevalence of treatment use and plateau in the elderly. On the other hand, systolic blood pressure continues to risk throughout life while diastolic blood pressure reaches a plateau and decreases in late life, reflecting widening of pulse pressure likely related to progressive arterial stiffness. Random blood glucose was used for the Original cohort.

Vascular risk factors including SBP, hypertension, fasting blood glucose, and triglycerides increased with age. On the other hand, other measures including DBP, total cholesterol, and LDL also increased in age until age 50-59 and then progressively decreased (Figures 5, 6). Of note, treatment use for vascular risk factors increased with age and seemed to parallel the decrease in the factors noted above (Figures 5, 6).

### ePVS and other CSVD markers

Although the sample size for some of the MRI markers differed (WMHV n=3362, CMB n=2291), we observed that the burden of CSVD markers increased as the grades of ePVS increased in both brain regions (Table 2). We observed that mean WMH volume and prevalence of CMB and CBI increased while mean total brain volume decreased in both the CSO and BG as the burden of ePVS increased. The mixed score of CSO and BG showed similar relation to other CSVD markers where marker severity increased with higher burden of ePVS.

**Table 2:** Association of ePVS with other cerebral small vessel disease (CSVD) markers.

		**ePVS Grade**										
		**Centrum Semiovale**				**Basal Ganglia**				**Mixed Centrum Semiovale-Basal Ganglia**		
		**I**	**II**	**III**	**IV**	**I**	**II**	**III**	**IV**	**0**	**1**	**2**
CSVD Markers	White matter hyperintensity volume, mean (SD), N=3362	0.91 (1.41)	2.7 (5.02)	8.51 (11.28)	10.62 (12.84)	1.14 (1.83)	3.12 (5.47)	10.52 (11.43)	24.39 (23.41)	1.52 (2.66)	7.23 (9.57)	13.82 (15.08)
	Total cranial to brain volume ratio, mean (SD), N=4101	78.46 (2.33)	76.6 (2.94)	74.65 (2.79)	73.88 (2.81)	78.1 (2.43)	76.38 (3.08)	73.95 (2.69)	72.91 (2.19)	77.62 (2.74)	74.7 (2.78)	73.74 (2.67)
	CMB presence, N (%), N=2291	44 (4)	50 (5)	15 (7)	11 (17)	49 (4)	54 (6)	16 (16)	1 (20)	91 (5)	15 (7)	14 (18)
	CBI presence, N (%), N=4101	53 (3)	157 (9)	107 (17)	42 (26)	75 (4)	172 (9)	95 (26)	17 (35)	175 (6)	107 (16)	77 (28)

ePVS were graded with scores I-IV to assign severity. High ePVS burden was defined as grades III-IV within each region. The mixed score represents participants with the following categories: no high burden of ePVS in the basal ganglia or the centrum Semiovale (grade 0), high burden in only one of these regions (grade 1), and high burden in both regions (grade 2).

### Multivariable analysis for the association of vascular risk factors and ePVS burden

Systolic and diastolic blood pressure were strongly associated with high ePVS burden in the CSO and BG, and the mixed CSO-BG score (Table 3). Similarly, hypertension was significantly associated with high ePVS burden in the CSO (OR: 1.66; 95% CI: 1.37, 2.01), BG (OR: 1.60; 95% CI: 1.32, 1.95) and in the mixed regions (OR: 1.49; 95% CI: 1.17, 1.90) (Table 3). The relation of hypertension category seemed to have a dose effect relation with ePVS burden in the CSO: we observed significant associations of prehypertension with increased ePVS burden (OR: 1.21; 95% CI: 1.05, 1.40) and stronger effect size with stage I hypertension or higher (OR: 1.49; 95% CI: 1.29, 1.72) (Table 3). Hypertension treatment use was also associated with high ePVS burden in the CSO and BG, but not the mixed region. Current smoking was associated with ePVS in the CSO (OR: 1.36; 95% CI: 1.07, 1.72), BG (OR:1.29; 95% CI: 1.03, 1.62) and mixed CSO-BG score (OR:1.42; 95% CI: 1.03, 1.98). A modest borderline association was seen between BMI and high ePVS burden in the CSO and BG.

**Table 3:** Association of vascular risk factors with high ePVS burden.

	**Centrum Semiovale**	**Basal Ganglia**	**Centrum Semiovale, Basal Ganglia - Mixed**
	**OR (95% CI)**	**OR (95% CI)**	**OR (95% CI)**
Age (years)	1.07 (1.06, 1.08) **	1.07 (1.06, 1.08) **	1.09 (1.08, 1.11) **
Men vs. Women	1.00 (0.88, 1.14)	1.08 (0.94, 1.24)	0.89 (0.74, 1.06)
Systolic blood pressure (mm Hg) ‡	1.21 (1.13, 1.30) **	1.26 (1.17, 1.36) **	1.18 (1.07, 1.29) *
Diastolic blood pressure (mm Hg) ‡	1.13 (1.06, 1.21) *	1.11 (1.03, 1.18) *	1.17 (1.07, 1.28) *
Hypertensive	1.66 (1.37, 2.01) **	1.60 (1.32, 1.95) **	1.49 (1.17, 1.90) *
Pre-hypertensive §	1.21 (1.05, 1.40) *	1.12 (0.97, 1.29)	1.16 (0.95, 1.42)
Normal	1.00 (Ref)	1.00 (Ref)	1.00 (Ref)
Hypertension Stage 1 or higher†	1.49 (1.29, 1.72) **	1.48 (1.28, 1.71) **	1.40 (1.16, 1.69) *
Current smoker	1.36 (1.07, 1.72) *	1.29 (1.03, 1.62) *	1.42 (1.03, 1.98) *
Total cholesterol (mg/dL) †	1.05 (0.99, 1.12)	0.97 (0.91, 1.04)	1.08 (0.99, 1.18)
LDL cholesterol (mg/dL) ||	1.05 (0.99, 1.12)	0.97 (0.91, 1.04)	1.03 (0.95, 1.13)
HDL cholesterol (mg/dL) #	0.93 (0.87, 1.00)	0.96 (0.89, 1.03)	1.00 (0.91, 1.10)
Diabetes	1.23 (0.99, 1.52)	1.11 (0.90, 1.38)	0.98 (0.76, 1.28)
Body Mass Index (BMI) †	1.05 (0.98, 1.12)	1.04 (0.97, 1.11)	0.96 (0.88, 1.06)
Hypertension Medication Use	1.24 (1.06, 1.44) *	1.39 (1.19, 1.61) *	1.19 (0.98, 1.43)
Lipid lowering Medication Use	0.93 (0.80, 1.08)	1.12 (0.96, 1.30)	0.93 (0.77, 1.11)

Models are adjusted for age, sex, time interval between examination and brain MRI, and FHS cohort. High ePVS burden was defined as grades III and IV within each region. The mixed score reflects participants with no high ePVS burden in neither the Basal Ganglia or Centrum Semiovale (grade 0) high ePVS burden in either the Basal Ganglia or the Centrum Semiovale (grade 1), and high burden in both the Centrum Semiovale and the Basal Ganglia (grade 2).

†For continuous variables except age, odds ratios represent increase per one standard deviation change.

‡Normal blood pressure is defined as systolic blood pressure (SBP) <120 mmHg and diastolic blood pressure (DBP) <80 mmHg.

§Prehypertension is defined as SBP of 120-139 mmHg or DBP 80-89mmHg. Hypertension is defined as SBP ≥140 mmHg or DBP ≥90 mm Hg. Blood pressure categories are defined irrespective of hypertension treatment status. Hypertension Stage 1 is defined as SBP of 130-139 mmHg or DBP of 80-89 mmHg.

*(p<0.05).

**p<0.0001.

| |LDL cholesterol is defined as low density lipoproteins.

#HDL cholesterol is defined as high density lipoproteins.

## DISCUSSION

We investigated the age- and sex-specific prevalence of ePVS in a large community-based sample of asymptomatic individuals and their relation to aging, individual vascular risk factors, and the use of commonly prescribed medications for cardiovascular prevention. The main findings of our study were the strong associations of age with ePVS burden, and characterization of changes in ePVS prevalence across age groups representing the entire life span. Some ePVS were observed in all participants, but high burden across brain regions was only noted after the fifth decade. We also observed that other CSVD markers increased in severity with higher burden of ePVS grades. Similarly, vascular risk factor burden increased with age and was associated with ePVS severity. The prevalence of ePVS increased with age in both men and women, but no sex differences were observed. Of note, in the Three-City (3C)-Dijon Magnetic Resonance Imaging Study [17], men appeared to have more BG-PVS than woman, although this has not been replicated in different studies [18]. Modifiable risk factors including hypertension, systolic and diastolic blood pressure, and current smoker status were associated with higher ePVS burden in the BG, CSO and mixed brain regions. Hypertension stage showed a dose effect relation with ePVS burden, with significant associations in the pre-hypertensive stage only in the CSO while the associations between hypertension stage I and higher were stronger and significant in the CSO, BG, and mixed regions. While hypertension treatment use was also associated with ePVS burden, this finding likely reflects bias by indication, where individuals using hypertension medications are more likely to have chronic and severe hypertension, in turn associated with ePVS burden, and importantly does not negate the benefits of hypertension medication use in prevention of cardiovascular events.

The age at which high ePVS burden increases (between 50 and 60 years) coincides with the age at which other studies have shown progression of measures of vascular injury such as arterial stiffness [19, 20], hypoperfusion [19, 21] and endothelial dysfunction [19], supporting the view that aging is a systemic process, and brain processes parallel systemic changes. Our results suggest that vascular risk factors, through their effect on microvasculature, ultimately might play a role in glymphatic dysfunction represented by ePVS, and although regional variations in the associations were seen, the relations with the mixed score suggest that vascular risk factors are related to ePVS burden across the brain.

The prevalence observed in the present study is in line with the reported prevalence in previous studies using similar methods for ePVS rating [22]. The association of ePVS with vascular risk factors, mainly hypertension, has been described in prior studies which demonstrated that low compliance with blood-pressure lowering drugs in patients with more severe ePVS [23, 24]. It is likely that vascular risk factor exposure over long periods of time lead to impaired perivascular drainage (glymphatic dysfunction) via multiple mechanisms, some of which may occur as a result of the aging process as well. For instance, vascular risk factors have been related to blood brain barrier disruption and increased in permeability, which has also been related to aging, with increased permeability in older individuals, but even more significantly in those with vascular dementia (VD) or Alzheimer’s disease (AD) [25]. Promotion of a microvascular inflammatory process [26, 27] and endothelial dysfunction [27] are also potential mechanism by which vascular risk factors may lead to glymphatic dysfunction and CSVD leading to enlargement of the perivascular spaces. The aging process has also been previously linked these processes [28]. Increased diastolic and systolic blood pressure, and hypertension stage 1 or higher showed associations with the prevalence of deep (BG), lobar (CSO) and mixed location ePVS. Our findings support the presumption that ePVS may reflect ongoing neurovascular unit dysfunction [19]. A higher burden of EPVS may signal increased ongoing neurodegeneration and vascular injury, both of which are part of the aging process. Previous small studies have shown an association with ambulatory systolic blood pressure and severe ePVS in the BG but not the CSO, which might indicate additional pathogenic mechanisms at interplay [29]. Of note, the strongest risk factor for CAA is also increased age, providing another potential mechanism on our observed associations [30].

The higher presence of other CSVD markers with higher ePVS burden is not surprising given the shared risk factors with other manifestations of CSVD. Future studies are needed to clarify the independent contribution of each CSVD marker vs assessments of the overall burden of CSVD in relation to aging and adverse neurological outcomes.

The present study has several strengths including the rating of brain MRI for PVS by trained readers and blinded to clinical data, the large unselected community- based sample which includes both men and women and the wide age range of participants. An important limitation is that Framingham Heart Study participants are of predominantly white, European descent, thus our findings apply to similar populations. We have also somewhat deviated from the rating method for PVS on axial slides, in an effort to increase our sample size and reduce the risk of bias by excluding participants from our studies. We hence rated PVS on coronal T2-MRI on a subset of our sample, using a method which we validated in-house and which provides a rating protocol that can be potentially used in other studies with similar technical issues. Further MRI-based studies within the FHS are currently underway to test different research hypothesis in the field of PVS.

## CONCLUSIONS

Our findings suggest that ePVS may also be considered a subclinical marker of aging and bring attention to their close relation and interplay with vascular risk factors, whose prevalence also rises with age in parallel to ePVS burden. The associations of high ePVS burden (and presumed glymphatic dysfunction) with increased blood pressure in particular, may begin earlier than previously thought, in pre-hypertension stages. Clinical trials would be needed to assess whether early treatment may prevent adverse cognitive consequences and stroke in individuals with high ePVS burden. Our study expands our understanding of ePVS in the context of aging and will provide the basis for future studies exploring PVS and other biomarker associations, as well as clinical correlates and outcomes in the Framingham Heart study.
